# Deep Transfer Learning Using Real-World Image Features for Medical Image Classification, with a Case Study on Pneumonia X-ray Images

**DOI:** 10.3390/bioengineering11040406

**Published:** 2024-04-20

**Authors:** Chanhoe Gu, Minhyeok Lee

**Affiliations:** 1 Department of Intelligent Semiconductor Engineering, Chung-Ang University, Seoul 06974, Republic of Korea; kum0100@cau.ac.kr; 2 School of Electrical and Electronics Engineering, Chung-Ang University, Seoul 06974, Republic of Korea

**Keywords:** deep learning, transfer learning, medical image analysis, X-ray images, pneumonia classification, convolutional neural networks, feature extraction, 68T27

## Abstract

Deep learning has profoundly influenced various domains, particularly medical image analysis. Traditional transfer learning approaches in this field rely on models pretrained on domain-specific medical datasets, which limits their generalizability and accessibility. In this study, we propose a novel framework called real-world feature transfer learning, which utilizes backbone models initially trained on large-scale general-purpose datasets such as ImageNet. We evaluate the effectiveness and robustness of this approach compared to models trained from scratch, focusing on the task of classifying pneumonia in X-ray images. Our experiments, which included converting grayscale images to RGB format, demonstrate that real-world-feature transfer learning consistently outperforms conventional training approaches across various performance metrics. This advancement has the potential to accelerate deep learning applications in medical imaging by leveraging the rich feature representations learned from general-purpose pretrained models. The proposed methodology overcomes the limitations of domain-specific pretrained models, thereby enabling accelerated innovation in medical diagnostics and healthcare. From a mathematical perspective, we formalize the concept of real-world feature transfer learning and provide a rigorous mathematical formulation of the problem. Our experimental results provide empirical evidence supporting the effectiveness of this approach, laying the foundation for further theoretical analysis and exploration. This work contributes to the broader understanding of feature transferability across domains and has significant implications for the development of accurate and efficient models for medical image analysis, even in resource-constrained settings.

## 1. Introduction

Deep learning has profoundly transformed various domains, particularly in the field of medical image analysis [1,2,3]. Convolutional Neural Networks (CNNs) have demonstrated exceptional performance in automatically extracting hierarchical features from images, leading to significant advancements in applications such as object recognition, image segmentation, and medical diagnostics [4,5,6,7,8].

Transfer learning has emerged as a fundamental technique in deep learning, allowing models to leverage knowledge acquired from source tasks to improve performance on target tasks [9,10,11]. This approach commonly employs pretrained models initially trained on large-scale datasets such as ImageNet as feature extractors for related tasks. These pretrained models capture a wide range of features, from low-level edges and textures to high-level semantic information, which can be effectively transferred to downstream tasks.

However, the application of transfer learning in the context of medical image analysis presents unique challenges. Medical images such as X-rays, Computed Tomography (CT) scans, and Magnetic Resonance Imaging (MRI) exhibit distinct characteristics compared to natural images. These images are typically grayscale, have high resolution, and contain intricate anatomical structures and pathological patterns [12,13,14]. Consequently, the direct application of pretrained models from natural image domains to medical image tasks may not always yield optimal results.

To address this challenge, current approaches in medical image transfer learning often rely on domain-specific pretrained models. These models are trained on large-scale medical image datasets, which capture the unique characteristics of medical images. While these domain-specific pretrained models have shown promising results, they have limitations in terms of accessibility and generalizability. The availability of large-scale annotated medical image datasets is limited, and the development of domain-specific pretrained models requires substantial computational resources and expertise.

In this paper, we propose a novel approach to transfer learning for medical image analysis, which we term “real-world feature transfer learning”. Our approach leverages pretrained models from general image domains such as ImageNet as feature extractors for medical image tasks. We hypothesize that the features learned by these models, despite being derived from natural images, can provide meaningful representations for medical images. By utilizing readily available pretrained models, our approach aims to overcome the limitations of domain-specific pretrained models and enable more accessible and efficient transfer learning for medical image analysis.

To validate our approach, we focus on the task of pneumonia detection using chest X-ray images. Pneumonia is a common respiratory infection that affects millions of people worldwide and is a leading cause of mortality, particularly in children and the elderly [15]. Accurate and timely diagnosis of pneumonia is crucial for effective treatment and patient management. Chest X-ray imaging is the primary diagnostic tool for pneumonia; however, the interpretation of these images can be challenging, even for experienced radiologists [16].

We conducted extensive experiments to evaluate the effectiveness of real-world feature transfer learning for pneumonia detection. We employed various conventional CNN architectures, including ResNet [17] and DenseNet [18], pretrained on the ImageNet dataset. To adapt these models to the grayscale nature of chest X-ray images, we propose a simple yet effective technique of replicating the grayscale channel to form a three-channel input compatible with the pretrained models.

Our experimental results demonstrate that real-world-feature transfer learning achieves superior performance compared to training models from scratch on the pneumonia detection task. The pretrained models exhibited faster convergence along with higher accuracy, precision, recall, and F1 scores. These findings suggest that the features learned from natural images can indeed be effectively transferred to medical image domains, even when the source and target domains have significant differences.

The significance of our work lies in its potential to democratize deep learning for medical image analysis. By leveraging readily available pretrained models from general image domains, our approach reduces the reliance on domain-specific pretrained models and large-scale annotated medical image datasets. This can facilitate the development of accurate and efficient models for various medical image analysis tasks even in resource-constrained settings. Furthermore, our approach opens up new avenues for exploring the transferability of features across different domains and modalities, potentially leading to more generalized and robust deep learning models.

From a mathematical perspective, our work contributes to the understanding of feature representations and their transferability across domains. We formalize the concept of real-world feature transfer learning and provide a rigorous mathematical formulation of the problem. Our experimental results provide empirical evidence supporting the effectiveness of this approach, laying the foundation for further theoretical analysis and exploration.

The main contributions of our work are as follows:

- We introduce the concept of real-world feature transfer learning, which leverages pretrained models from general image domains for medical image analysis tasks.
- We propose a simple and effective technique for adapting pretrained models to grayscale medical images by replicating the grayscale channel.
- We conduct extensive experiments on the pneumonia detection task using chest X-ray images, demonstrating the superiority of real-world feature transfer learning over training models from scratch.
- We provide empirical evidence supporting the feasibility and effectiveness of transferring knowledge from natural image domains to medical image domains, opening up new possibilities for accessible and efficient transfer learning in medical image analysis.

## 2. Related Work

Transfer learning has gained significant attention in the deep learning community, particularly in the field of medical image analysis. The concept of transfer learning involves leveraging knowledge acquired from a source domain to improve performance on a target domain [19,20]. In the context of deep learning, transfer learning often involves using pretrained models trained on large-scale datasets as feature extractors or performing initialization for target tasks [21,22,23].

Recent studies have investigated the effectiveness of transfer learning for various medical image analysis tasks. Alzubaidi et al. [24] proposed a novel transfer learning approach for skin and breast cancer classification with medical images. They trained a deep convolutional neural network (DCNN) model on large unlabeled medical image datasets and then transferred the knowledge to train the model on a small amount of labeled medical images. Their approach showed significantly improved performance on both skin and breast cancer classification tasks compared to training from scratch. In the domain of chest X-ray analysis, transfer learning has been extensively explored in recent years. Rahman et al. [25] investigated the effectiveness of transfer learning for pneumonia detection in chest X-rays. They evaluated various pretrained CNN architectures, such as AlexNet, ResNet18, DenseNet201, and SqueezeNet, and found that fine-tuning these models led to improved performance compared to training from scratch. Chouhan et al. [26] proposed a novel transfer learning approach for pneumonia detection using an ensemble of pretrained CNN models. Their approach achieved state-of-the-art performance on a public dataset, outperforming individual CNN models.

While these studies have shown promising results, they primarily focus on transfer learning within the medical domain, using pretrained models that have been trained on medical image datasets. In contrast, our work explores the feasibility of transfer learning from general image domains to medical image domains by leveraging pretrained models from datasets such as ImageNet.

The concept of transferring knowledge from general image domains to medical image domains has gained attention in recent studies. Raghu et al. [27] investigated the transferability of features from natural image datasets to medical image tasks. They found that pretrained models from ImageNet can be effectively adapted to medical image classification tasks, achieving comparable performance to models trained from scratch on medical datasets.

Building upon these findings, our work aims to provide a comprehensive analysis of the effectiveness of real-world feature transfer learning for medical image analysis, focusing on the task of pneumonia detection in chest X-rays. We extend the existing literature by investigating the transferability of features from general image domains to the specific domain of chest X-ray analysis, and propose a simple yet effective technique for adapting pretrained models to grayscale medical images.

In addition to transfer learning, our work is related to the broader field of deep learning for medical image analysis. Deep learning has revolutionized the field of medical image analysis, enabling the development of automated systems for various tasks, including classification, detection, and segmentation [2,28]. CNNs have become the dominant architecture in medical image analysis thanks to their ability to learn hierarchical features directly from raw image data [29,30].

Several deep learning-based approaches have been proposed for pneumonia detection in chest X-rays in recent years. Ayan and Ünver [31] developed a deep learning model using a custom CNN architecture for pneumonia detection. They achieved high accuracy and sensitivity on a public dataset, demonstrating the effectiveness of deep learning for this task. Our work extends these studies by specifically focusing on the transfer learning aspect and exploring the feasibility of leveraging pretrained models from general image domains for pneumonia detection. We provide a comprehensive analysis of different CNN architectures and their performance when utilized within a real-world feature transfer learning framework.

## 3. Background

Transfer learning is a machine learning technique that leverages knowledge gained from solving one problem and applies it to a different but related problem [19]. The goal of transfer learning is to improve learning performance in the target domain utilizing the knowledge learned from the source domain. This section provides a mathematical formulation of transfer learning and its key concepts.

### 3.1. Problem Formulation

**Definition 1.**  *A domain D consists of a feature space X and a marginal probability distribution P(X), where $X=\{x_{1},\ldots ,x_{n}\}\in X$.*

**Definition 2.**  *Given a domain D={X,P(X)}, a task T consists of a label space Y and a conditional probability distribution P(Y|X), which is learned from the training data $\left\{\left(x_{i},y_{i}\right)\right\}$, where $x_{i}\in X$ and $y_{i}\in Y$.*

**Definition 3.**  *Given a source domain $D_{S}$ and corresponding source task $T_{S}$ along with a target domain $D_{T}$ and corresponding target task $T_{T}$, transfer learning aims to improve the learning of the targeted conditional probability distribution $P(Y_{T}|X_{T})$ in $D_{T}$ using the knowledge learned from $D_{S}$ and $T_{S}$, where $D_{S}\neq D_{T}$ or $T_{S}\neq T_{T}$.*

### 3.2. Categories of Transfer Learning

Transfer learning can be categorized into three main settings based on the differences between the source and target domains and the tasks involved [19].

#### 3.2.1. Inductive Transfer Learning

**Definition 4.**  *In inductive transfer learning, the target task is different from the source task, regardless of whether or not the source and target domains are the same. In this setting, the target domain labeled data are available, and the goal is to improve the target task performance using the knowledge learned from the source domain.*

Given a source domain $D_{S}$ and corresponding source task $T_{S}$ along with a target domain $D_{T}$ and corresponding target task $T_{T}$, inductive transfer learning aims to improve the learning of the targeted conditional probability distribution $P(Y_{T}|X_{T})$ using the knowledge learned from $D_{S}$ and $T_{S}$, where $T_{S}\neq T_{T}$.

#### 3.2.2. Transductive Transfer Learning

**Definition 5.**  *In transductive transfer learning, the source and target tasks are the same, while the source and target domains are different. In this setting, the target domain labeled data are not available, and the goal is to improve the target task performance using the knowledge learned from the source domain.*

Given a source domain $D_{S}$ and corresponding source task $T_{S}$ along with a target domain $D_{T}$ and corresponding target task $T_{T}$, transductive transfer learning aims to improve the learning of the target conditional probability distribution $P(Y_{T}|X_{T})$ using the knowledge learned from $D_{S}$ and $T_{S}$, where $D_{S}\neq D_{T}$ and $T_{S}=T_{T}$.

#### 3.2.3. Unsupervised Transfer Learning

**Definition 6.**  *In unsupervised transfer learning, the target task is different from but related to the source task, and no labeled data are available in either the source or target domains. The goal is to improve the target task performance using the knowledge learned from the source domain in an unsupervised manner.*

Given a source domain $D_{S}$ and corresponding source task $T_{S}$ along with a target domain $D_{T}$ and corresponding target task $T_{T}$, unsupervised transfer learning aims to improve the learning of the targeted conditional probability distribution $P(Y_{T}|X_{T})$ using the knowledge learned from $D_{S}$ and $T_{S}$, where $T_{S}\neq T_{T}$ and where no labeled data are available in either $D_{S}$ or $D_{T}$.

### 3.3. Transfer Learning in Deep Neural Networks

Deep neural networks have shown remarkable success in various machine learning tasks, particularly in computer vision and natural language processing [32,33]. Transfer learning in deep neural networks typically involves using a pretrained model trained on a large-scale source dataset as a starting point for a target task with limited labeled data.

**Definition 7.**  *Let $f_{S}:X_{S}\to Y_{S}$ be a pretrained deep neural network model for source task $T_{S}$, parameterized by $\theta_{S}$. The goal of transfer learning in deep neural networks is to learn a target model $f_{T}:X_{T}\to Y_{T}$, parameterized by $\theta_{T}$, by leveraging the knowledge learned from $f_{S}$.*

The transfer learning process in deep neural networks can be formalized as follows:

(1) $$\begin{array}{cc} \theta_{T}^{\left(0\right)} & =\theta_{S}^{*}, \end{array}$$

(2) $$\begin{array}{cc} \theta_{T}^{*} & =\arg\min_{\theta_{T}}L_{T}\left(f_{T}\left(X_{T};\theta_{T}\right),Y_{T}\right), \end{array}$$

where $\theta_{S}^{*}$ represents the optimal parameters of the pretrained source model $f_{S}$, $\theta_{T}^{\left(0\right)}$ represents the initial parameters of the target model $f_{T}$, $L_{T}$ is the loss function for the target task, and $\theta_{T}^{*}$ represents the optimal parameters of the target model after fine-tuning.

**Remark 1.**  *The pretrained source model $f_{S}$ can be used in various ways for transfer learning, such as:*

- *Using $f_{S}$ as a fixed feature extractor and training a new classifier on top of the extracted features for the target task.*
- *Fine-tuning the entire network $f_{S}$ using the target task data, allowing the pretrained parameters to adapt to the target domain.*
- *Freezing some layers of $f_{S}$ and fine-tuning the remaining layers for the target task, balancing the knowledge transfer and adaptation to the target domain.*

The choice of transfer learning strategy depends on factors such as the size of the target dataset, the similarity between the source and target domains, and the available computational resources.

### 3.4. Transfer Learning as a Foundation for Segmentation and Object Detection

The paradigm of transfer learning, in which pretrained models serve as the backbone for high-level vision tasks, has become a fundamental approach in the fields of segmentation and object detection [34,35,36,37,38,39]. This section provides a rigorous mathematical formulation of how this methodology is adapted for these specific tasks.

#### 3.4.1. Segmentation

**Definition 8.**  *Let X be the input space of images and let Y be the output space of pixel-wise class labels. The goal of image segmentation is to learn a mapping function $f_{seg}:X\to Y$ that assigns a class label to each pixel in an image.*

**Assumption 1.**  We assume the existence of a pretrained model, represented by parameters $\theta^{*}$, that has learned meaningful and generalizable representations from a large-scale dataset $D_{S}$.

**Proposition 1.**  The mapping function $f_{seg}$ can be decomposed into two components: the pretrained backbone model, parameterized by $\theta^{*}$, and additional segmentation-specific layers, parameterized by ψ.

(3) $$\begin{array}{c} f_{seg}\left(x;\theta^{*},\psi \right)=\psi \circ \theta^{*}\left(x\right)\;\forall x\in X \end{array}$$

**Definition 9.**  Let $x_{i}$ denote a pixel in an image x∈X and let $y_{i}$ be its corresponding segmentation label. The objective of the segmentation task is to learn the optimal parameters $\psi_{seg}^{*}$ that minimize the expected loss over the target dataset $D_{T}$:

(4) $$\psi_{seg}^{*}=\arg\min_{\psi }E_{x,y∼D_{T}}\left[\frac{1}{\left|X\right|}\sum_{i=1}^{\left|X\right|}L\left(y_{i},f_{seg}\left(x_{i};\theta^{*},\psi \right)\right)\right]$$

where *L* is a suitable loss function, such as the cross-entropy loss, and |X| denotes the total number of pixels in the image.

#### 3.4.2. Object Detection

**Definition 10.**  *Let B be the space of bounding boxes and let C be the space of object classes. The goal of object detection is to learn a mapping function $f_{det}:X\to B\times C$ that predicts a set of bounding boxes and their associated class labels for objects in an image.*

**Proposition 2.**  Similar to segmentation, the mapping function $f_{det}$ can be decomposed into the pretrained backbone model, parameterized by $\theta^{*}$, and additional detection-specific layers, parameterized by ψ.

(5) $$\begin{array}{c} f_{det}\left(x;\theta^{*},\psi \right)=\psi \circ \theta^{*}\left(x\right)\;\forall x\in X \end{array}$$

**Definition 11.**  Let $y_{b,c}$ denote the ground-truth bounding boxes and their associated class labels for an image x∈X. The objective of the object detection task is to learn the optimal parameters $\psi_{det}^{*}$ that minimize the expected loss over the target dataset $D_{T}$:

(6) $$\psi_{det}^{*}=\arg\min_{\psi }E_{x,y∼D_{T}}\left[L(y_{b,c},f_{det}\left(x;\theta^{*},\psi \right))\right]$$

where *L* is a loss function that typically includes terms for both the bounding box coordinates and the class labels.

**Remark 2.**  *In practice, it is common to fine-tune the entire network, both θ and ψ, on the target task dataset in order to achieve better performance. This approach differs from conventional transfer learning, where only the target task parameters ψ are fine-tuned while keeping the source task parameters θ fixed.*

**Proposition 3.**  *The fine-tuning process for segmentation and object detection can be formulated as follows:*

(7) $$\begin{array}{cc} \left(\theta_{seg}^{*},\psi_{seg}^{*}\right) & =\arg\min_{\theta ,\psi }E_{x,y∼D_{T}}\left[\frac{1}{\left|X\right|}\sum_{i=1}^{\left|X\right|}L\left(y_{i},f_{seg}\left(x_{i};\theta ,\psi \right)\right)\right] \end{array}$$

(8) $$\begin{array}{cc} \left(\theta_{det}^{*},\psi_{det}^{*}\right) & =\arg\min_{\theta ,\psi }E_{x,y∼D_{T}}\left[L(y_{b,c},f_{det}\left(x;\theta ,\psi \right))\right] \end{array}$$

where $\theta_{seg}^{*}$ and $\theta_{det}^{*}$ denote the respective fine-tuned backbone parameters for segmentation and object detection.

**Remark 3.**  *The fine-tuning process allows the pretrained backbone to adapt its learned representations to the specific characteristics of the target task, leading to improved performance compared to using fixed backbone parameters.*

### 3.5. Examples of Transfer Learning

Transfer learning has become a fundamental technique in deep learning, particularly in the field of computer vision. The main idea behind transfer learning is to leverage the knowledge gained from a source task and apply it to a related target task, thereby improving the performance and efficiency of learning on the target task. This is especially useful when the target task has limited labeled data, as is often the case in medical image analysis.

In the context of deep learning, transfer learning typically involves using a pretrained neural network model as a starting point for the target task. The pretrained model is usually trained on a large-scale dataset, such as ImageNet, which contains millions of labeled images spanning a wide range of object categories. During the pretraining phase, the model learns a rich set of features that capture various aspects of the images, including low-level edges and textures as well as high-level semantic information.

When applying transfer learning to a target task, such as medical image classification, the pretrained model is used as a feature extractor. The weights of the pretrained model are initialized with the learned values from the source task, and the model is then fine-tuned on the target task dataset. Fine-tuning involves updating the weights of the pretrained model using the labeled data from the target task, allowing the model to adapt its learned features to the specific characteristics of the target domain.

There are several strategies for fine-tuning a pretrained model. One common approach is to freeze the weights of the early layers of the network, which capture general low-level features, and only fine-tune the later layers, which capture more task-specific high-level features; this allows the model to retain the knowledge learned from the source task while adapting to the target task. Another approach is to fine-tune the entire network, allowing all of the weights to be updated based on the target task data. The choice of fine-tuning strategy depends on factors such as the size of the target dataset, the similarity between the source and target domains, and the available computational resources.

Transfer learning has been successfully applied to a wide range of computer vision tasks, including image classification, object detection, and semantic segmentation. For example, in image classification, transfer learning has been used to improve the performance of models trained on limited labeled data by leveraging pretrained models from large-scale datasets. In object detection, transfer learning has been used to adapt pretrained models trained on general object detection datasets to specific domains, including medical imaging, by fine-tuning the models on domain-specific data. Similarly, in semantic segmentation, transfer learning has been used to improve the performance of models by initializing them with weights learned from pretraining on large-scale datasets and then fine-tuning them on the target task data.

The effectiveness of transfer learning in deep learning can be attributed to several factors. First, deep neural networks have the ability to learn hierarchical features that capture different levels of abstraction in the data. By pretraining on a large-scale dataset, the model learns a rich set of features that can be transferred to related tasks. Second, the use of transfer learning allows for more efficient learning on the target task, as the model does not need to learn the features from scratch. This is particularly important when the target task has limited labeled data, as the model can leverage the knowledge learned from the source task to improve its performance. Finally, transfer learning can help to reduce overfitting on the target task, as the pretrained model has already learned a robust set of features that generalize well to related tasks.

In the context of medical image analysis, transfer learning has been widely adopted to address the challenges of limited labeled data and the need for efficient and accurate models. Medical imaging datasets are often small and expensive to annotate, making it difficult to train deep learning models from scratch. By leveraging pretrained models from large-scale datasets, transfer learning allows for the development of accurate models for medical image analysis tasks, even with limited labeled data. This has led to significant improvements in the performance of deep learning models for tasks such as medical image classification, segmentation, and detection.

Despite the success of transfer learning in medical image analysis, there are still challenges and opportunities for further research. One challenge is the domain shift between the source and target tasks, which can limit the effectiveness of transfer learning. This is particularly relevant when transferring knowledge from natural image datasets to medical image datasets, as there can be significant differences in the characteristics of the images and the underlying data distributions. To address this challenge, techniques such as domain adaptation and unsupervised domain adaptation have been proposed to align the feature spaces of the source and target domains and improve the transferability of the learned features.

## 4. Proposition: Similarity of Learned Features in Conventional and Medical Images

This section presents a mathematical framework to demonstrate the similarity between the features learned by deep learning models from conventional images and medical images. We establish this proposition through a series of definitions, assumptions, and mathematical formulations.

**Definition 12.**  *Let $X_{C}$ and $X_{M}$ denote the input spaces of conventional images and medical images, respectively. We define a feature extraction function ϕ:X→F, where F is the feature space and $X=X_{C}\cup X_{M}$.*

**Assumption 2.**  *We assume that the feature extraction function ϕ is a deep learning model parameterized by θ which has been trained on a large dataset of conventional images $D_{C}={\left\{\left(x_{i},y_{i}\right)\right\}}_{i=1}^{N_{C}}$, where $x_{i}\in X_{C}$ and $y_{i}$ is the corresponding label.*

**Definition 13.**  *Let $F_{C}$ and $F_{M}$ be the feature spaces corresponding to conventional and medical images, respectively, such that $F_{C}=\phi \left(X_{C}\right)$ and $F_{M}=\phi \left(X_{M}\right)$.*

**Proposition 4.**  *The features learned by the deep learning model ϕ from conventional images are similar to the features of medical images, i.e., $F_{C}\approx F_{M}$.*

To prove this proposition, we introduce a measure of similarity between the feature spaces.

**Definition 14.**  *Let d:F×F→R be a distance function that quantifies the dissimilarity between two feature vectors. We define the average distance between the feature spaces $F_{C}$ and $F_{M}$ as*

(9) $$D\left(F_{C},F_{M}\right)=\frac{1}{|X_{C}\left|\right|X_{M}|}\sum_{x_{C}\in X_{C}}\sum_{x_{M}\in X_{M}}d\left(\phi \left(x_{C}\right),\phi \left(x_{M}\right)\right).$$

**Assumption 3.**  *We assume that the distance function d satisfies the properties of a metric, i.e., non-negativity, identity of indiscernibles, symmetry, and triangle inequality.*

**Lemma 1.**  *If the average distance between the feature spaces $F_{C}$ and $F_{M}$ is small, i.e., if $D(F_{C},F_{M})<\epsilon$ for some small ϵ>0, then the features learned from conventional images are similar to the features of medical images.*

**Proof.**  Let $x_{C}\in X_{C}$ and $x_{M}\in X_{M}$ be arbitrary conventional and medical images, respectively. From the definition of the average distance and the assumption that $D(F_{C},F_{M})<\epsilon$, we have

(10) $$\begin{array}{cc} d(\phi \left(x_{C}\right),\phi \left(x_{M}\right)) & \leq \sum_{x_{C}^{\prime }\in X_{C}}\sum_{x_{M}^{\prime }\in X_{M}}d\left(\phi \left(x_{C}^{\prime }\right),\phi \left(x_{M}^{\prime }\right)\right), \end{array}$$

(11) $$\begin{array}{cc} \; & =|X_{C}\left|\right|X_{M}|D\left(F_{C},F_{M}\right), \end{array}$$

(12) $$\begin{array}{cc} \; & <|X_{C}\left|\right|X_{M}|\epsilon . \end{array}$$

This implies that, for any pair of conventional and medical images, the distance between their feature representations is bounded by a small value proportional to ϵ; therefore, the features learned from conventional images are similar to the features of medical images. □

**Remark 4.**  *The choice of the distance function d is crucial for quantifying the similarity between feature spaces. Common choices include the Euclidean distance, cosine distance, and more advanced metrics such as the Fréchet Inception Distance (FID) [40].*

**Conjecture 1.**  *The similarity between the features learned from conventional images and the features of medical images can be further improved by fine-tuning the pretrained model ϕ on a small dataset of medical images $D_{M}={\left\{\left(x_{i},y_{i}\right)\right\}}_{i=1}^{N_{M}}$, where $x_{i}\in X_{M}$ and $y_{i}$ is the corresponding label.*

**Proposition 5.**  *Fine-tuning the pretrained model ϕ on the medical image dataset $D_{M}$ results in an updated feature extraction function $\phi^{\prime }$ that minimizes the average distance between the feature spaces $F_{C}$ and $F_{M}$.*

**Proof.**  The fine-tuning process can be formulated as an optimization problem:

(13) $$\theta^{*}=\arg\min_{\theta }\frac{1}{N_{M}}\sum_{i=1}^{N_{M}}L\left(y_{i},f\left(\phi^{\prime }\left(x_{i};\theta \right)\right)\right)$$

where *L* is a suitable loss function and *f* is a task-specific function (e.g., a classifier or segmentation model) that operates on the features extracted by $\phi^{\prime }$.

By minimizing the loss function on the medical image dataset, the fine-tuning process adapts the parameters θ to better capture the characteristics of medical images. As a result, the updated feature extraction function $\phi^{\prime }$ maps medical images to a feature space $F_{M}^{\prime }$ that is closer to the feature space $F_{C}$ of conventional images, i.e., $D\left(F_{C},F_{M}^{\prime }\right)<D\left(F_{C},F_{M}\right)$. □

## 5. Method

**Figure 1:**
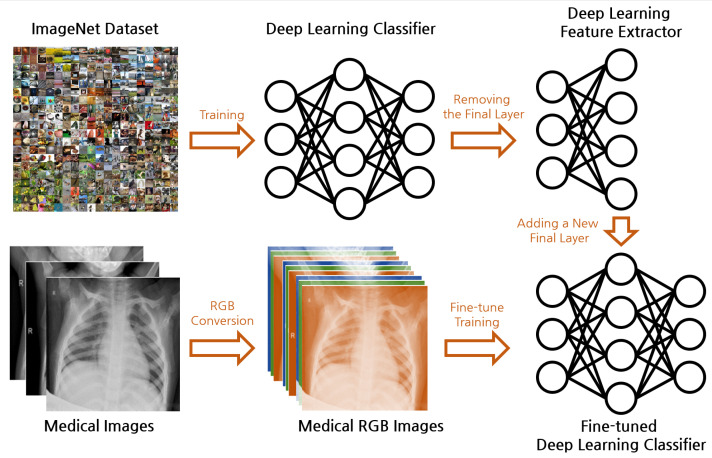
Overall framework of the proposed real-world feature transfer learning approach for medical image classification.

Figure 1 illustrates the overall framework of our proposed real-world feature transfer learning approach for medical image classification. Our methodology aims to reconcile the commonly held belief that transfer learning between general image datasets such as ImageNet and specialized medical image datasets such as X-ray images is challenging due to the disparity in their features. Typically, deep learning models trained on datasets such as ImageNet learn to recognize a plethora of real-world objects, such as the shape of animals, color of fruits, structure of machines, etc. In contrast, medical images are predominantly grayscale, and contain intricate patterns and details that represent health conditions and diseases. This substantial difference in features makes transfer learning a challenging task.

Further, a conventional deep learning model expects RGB images as input, necessitating the alteration of the model structure to accommodate grayscale images, which often complicates the transfer learning process. In our methodology, we propose an approach that circumvents these limitations and demonstrate the feasibility of transfer learning between these dissimilar domains.

### 5.1. Data Preparation

Medical images are generally grayscale, representing the information in a single color channel, whereas typical real-world images are composed of three color channels (RGB). In order to apply a pretrained model expecting three channels to medical images, we propose a simple yet effective approach: we replicate the grayscale channel three times to mimic the structure of an RGB image, ensuring the compatibility of the medical images with the pretrained model without requiring any modification to the model’s architecture.

(14) $$I_{RGB}=I_{gray}⊗\left[1,1,1\right]$$

Here, $I_{RGB}$ is the transformed RGB image, $I_{gray}$ is the original grayscale medical image, and [1,1,1] is the vector used to replicate the grayscale channel.

### 5.2. Model Architecture

Our model architecture is designed to leverage the power of transfer learning while adapting to the specific characteristics of medical image classification. The architecture consists of two primary components: a pretrained model serving as the backbone feature extractor, and an appended layer(s) for task-specific fine-tuning.

The pretrained model, denoted as F(x;θ,ϕ), is a variant of deep CNN that has been previously trained on a large-scale dataset such as ImageNet. This pretrained model has learned a rich set of features that capture the intricate patterns and structures present in natural images. By leveraging these learned features, the knowledge gained from the source domain can be effectively transferred to the target domain of medical image classification.

In our architecture, we utilize the pretrained model as a fixed feature extractor. This means that we freeze the weights of the pretrained model layers, represented by the parameters θ, during the fine-tuning process. By keeping these weights fixed, we preserve the valuable feature representations learned from the source domain and prevent overfitting to the limited labeled data in the target domain.

To adapt the pretrained model to the specific task of medical image classification, we append one or more layers, represented by G(x;θ,ψ), on top of the pretrained model. These appended layers are designed to learn the task-specific mapping between the extracted features and the corresponding class labels. The parameters of these appended layers, denoted as ψ, are trainable, and are fine-tuned using the labeled medical image data.

During the fine-tuning process, the weights of the appended layers are updated using the labeled medical image data. This allows the model to adapt to the specific characteristics and patterns present in the medical images. The fine-tuning process is typically performed using a smaller learning rate compared to the initial training of the pretrained model, as we want to preserve the knowledge gained from the source domain while gradually adapting to the target domain.

By leveraging the power of transfer learning through the use of a pretrained model as the backbone and fine-tuning the appended layers, our model architecture effectively captures the relevant features and patterns in medical images. This approach allows us to benefit from the knowledge gained from large-scale datasets while adapting to the specific requirements of medical image classification tasks.

### 5.3. Transfer Learning

We employ transfer learning, leveraging the knowledge acquired by the pretrained model from the source domain (general images) and applying it to the target domain (medical images); this technique assumes that the learned feature representations in the source task are beneficial to the learning in the target task.

As discussed in regard to related work, this is expressed mathematically as follows:

(15) $$\psi^{*}=\arg\min_{\psi }E_{x,y∼D_{T}}\left[L\left(y,G\left(x;\theta^{*},\psi \right)\right)\right].$$

In the above equation, $D_{T}$ represents the target domain data and $L(y,G\left(x;\theta^{*},\psi \right))$ is the loss function that we aim to minimize. The parameters ψ are then fine-tuned based on the medical image data.

### 5.4. Experimental Setup

Our study employed seven widely used pretrained models as the basis for transfer learning, each of which has demonstrated exceptional performance on the ImageNet dataset. We then tailored these models to our medical imaging task by freezing their convolutional layers and training a new fully connected layer that we appended at the end of each model. Below, we provide more detailed descriptions of each model used:

- **ResNet18 and ResNet50** [17]: The ResNet family of models are residual learning frameworks that provide an effective solution to the problem of training deep networks. They introduce the concept of skip connections, or shortcuts, that allow the gradient to be directly backpropagated to earlier layers. In our study, we used ResNet18 and ResNet50, which consist of 18 and 50 layers, respectively.
- **DenseNet161** [18]: DenseNet161 is a member of the DenseNet family, which connects each layer to every other layer in feed-forward fashion. This allows for feature reuse throughout the network, making the model more parameter-efficient. DenseNet161, the variant we used, consists of 161 layers.
- **ShuffleNet v2 (1.0×)** [41]: ShuffleNet is a highly efficient convolutional neural network designed for mobile devices. It introduces two novel operations, pointwise group convolution and channel shuffle, to reduce computation cost while maintaining model accuracy. We utilized version 2, with a scale factor of 1.0×, in our work.
- **MobileNet v2** [42]: Similar to ShuffleNet, MobileNet v2 is designed for mobile and embedded vision applications. It leverages inverted residuals and linear bottlenecks to create a lightweight yet performant architecture.
- **ResNeXt50 (32x4d)** [43]: ResNeXt50 is a member of the ResNeXt family, which offers a simple and scalable way to increase model capacity without a significant increase in computational cost. The variant we used, denoted as 32x4d, indicates that the network has a cardinality (number of parallel paths) of 32 and a width (number of channels) of 4.
- **Wide ResNet50-2** [44]: Wide ResNet is a variant of the ResNet models with wider convolutional layers instead of deeper ones. The version we used, Wide ResNet50-2, indicates that the network depth is 50 and the width is twice that of the original ResNet50.

Each model was fine-tuned using the Pneumonia X-ray dataset [45] with Binary Cross-Entropy (BCE) as the loss function. We adopted a training approach that preserves the learned features in the pretrained models by freezing the weights in the convolutional layers and only updating the weights in the newly appended fully connected layer.

### 5.5. Model Description

Each model was obtained from the model pretrained on ImageNet. We retained the entire architecture except for the last layer, which we replaced with a fully connected layer followed by a Sigmoid activation function. This modification was necessary in order to adjust the output size to match our binary classification task (Pneumonia/Normal). These models are each popular deep learning architectures that have shown excellent performance in image classification tasks.

### 5.6. Hyperparameters

The chosen hyperparameters for our experiments were an epoch size of 20 and a batch size of 64. These values were chosen based on previous studies and our preliminary experiments.

### 5.7. Data Augmentation

Data augmentation is crucial for training deep learning models, as it can reduce overfitting and improve the model’s generalization capability. We applied a series of random transformations, including resizing and cropping, color jittering, horizontal flipping, and rotation. In addition, we transformed the images to grayscale, as the original X-ray images are grayscale, and normalized them to have a mean of 0.5 and a standard deviation of 0.5.

### 5.8. Dataset

**Figure 2:**
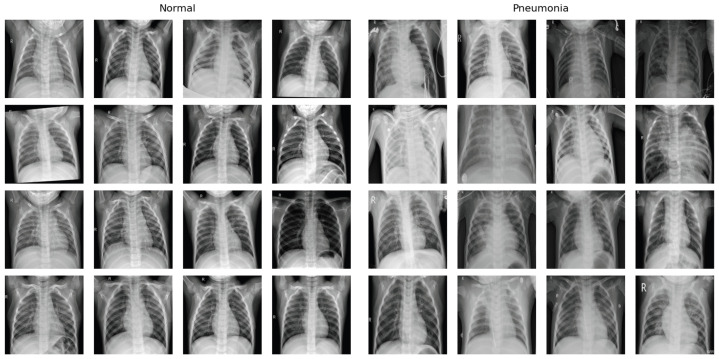
Example X-ray images of each class within the Pneumonia X-ray dataset. The images on the left showcase normal X-ray images and the images on the right showcase pneumonia images.

The Pneumonia X-ray dataset, which contains a total of 5863 X-ray images categorized into Pneumonia and Normal, was used for this study. The dataset is divided into training and testing sets, with 1341 normal and 3875 pneumonia images in the training set and 234 normal and 390 pneumonia images in the testing set. Large disparities in the distribution of classes represent a common challenge in medical datasets, which we addressed during the training phase by using class weights in the loss function. The sample distribution and example images of each class are displayed in Table 1 and Figure 2.

### 5.9. Evaluation Metrics

To assess the performance of our models, we utilized four key metrics: accuracy, precision, recall, and F1 score. These metrics provide a comprehensive understanding of model performance by considering the true positive, false positive, true negative, and false negative predictions made by the model.

The accuracy is defined as the proportion of correct predictions (both true positives and true negatives) out of all predictions.

(16) $$\mathrm{Accuracy}=\frac{TP+TN}{TP+TN+FP+FN}$$

where TP denotes true positives, TN denotes true negatives, FP denotes false positives, and FN denotes false negatives.

Precision (also known as positive predictive value) measures the proportion of correctly predicted positive observations out of the total predicted positives.

(17) $$\mathrm{Precision}=\frac{TP}{TP+FP}$$

Recall (or sensitivity) measures the proportion of correctly predicted positive observations out of the total actual positives.

(18) $$\mathrm{Recall}=\frac{TP}{TP+FN}$$

Finally, the F1 score is the weighted average (harmonic mean) of precision and recall, which helps to gauge the balance between precision and recall. It is particularly useful when the data class distribution is uneven.

(19) $$F1\;\mathrm{Score}=2\times \frac{\mathrm{Precision}\times \mathrm{Recall}}{\mathrm{Precision}+\mathrm{Recall}}$$

The above metrics were computed at the end of each epoch to provide a detailed evaluation of the model’s performance over time.

## 6. Results

### 6.1. Efficacy of Transfer Learning: Training Results

**Figure 3:**
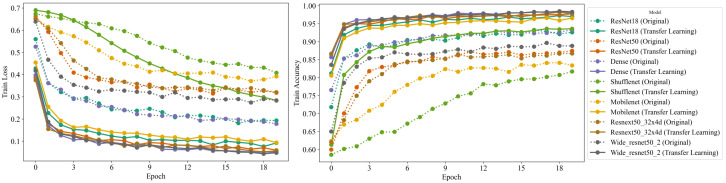
Graphical representation of the training loss and accuracy achieved by the models over time. The solid lines represent the real-world feature transfer learning models, while the dashed lines represent from-scratch training models. The rapid convergence of the proposed models demonstrates the feasibility and effectiveness of transferring learning from real-world images to medical images.

To validate the efficacy of our approach, we first analyze the results obtained during the training phase. Figure 3 presents a graphical representation of both the training loss and the accuracy achieved by the models over time. The rapid convergence of the models substantiates the premise that transfer learning from real-world images to medical images is indeed feasible and highly effective.

A comprehensive comparison of the different model architectures shows that all of the models except ShuffleNet surpassed 80% accuracy within a single epoch. This rapid ascent in accuracy reflects the efficiency of transfer learning and the ability of the pretrained models to generalize their learning from the source task (general image data) to the target task (medical image data). The adoption of features already learned by the models on a large-scale image dataset (ImageNet) evidently accelerates the learning process, leading to impressive early-stage performance.

Furthermore, we observed that all the models leveraging transfer learning significantly outperformed the regular models (without transfer learning) by reaching an accuracy above 90% after 20 epochs. In stark contrast, the regular models fell short of achieving a similar level of performance. This discrepancy underscores the considerable advantage conferred by transfer learning, particularly the ability to reuse the knowledge from general images, paving the way for high-performance models even in complex domains such as medical imaging.

Among the models we experimented with, ResNeXt50 and Wide ResNet50 performed exceptionally well in the early stages, both achieving over 95% training accuracy within the first few epochs. These models’ rapid convergence can be attributed to their deep and wide architectures and unique design principles (skip connections in ResNet), which evidently facilitate feature propagation and mitigate the problem of vanishing gradients.

Conversely, the ShuffleNet model, designed with a focus on computational efficiency, demonstrated a slower convergence rate compared to the other models, only reaching 80% accuracy after several epochs. Nevertheless, it is worth noting that while ShuffleNet trailed in early-stage accuracy, its design philosophy aims to maintain a balance between accuracy and computational efficiency, which is paramount in certain scenarios, particularly mobile and embedded applications.

These experiments yielded compelling evidence supporting the effectiveness of transfer learning from general image data to medical image data. The models leveraging transfer learning exhibited rapid convergence and high accuracy, underscoring the feasibility of our approach and its potential implications for the domain of medical image analysis.

### 6.2. Comparative Analysis of Models: Test Results

**Figure 4:**
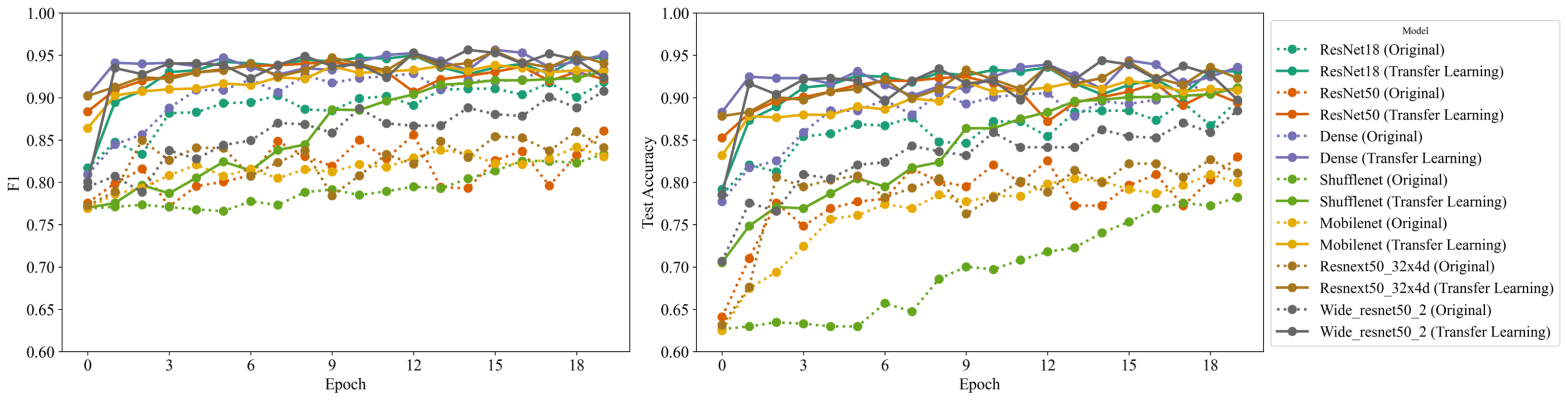
Comprehensive comparison between transfer learning models and models trained from scratch. The line graphs illustrate the performance metrics of test accuracy and F1 score for each model. The solid lines represent the real-world feature transfer learning models, while the dashed lines represent from-scratch training models. The comparison emphasizes the superiority of the transfer learning approach for handling medical image classification.

In order to more deeply investigate the performance of our transfer learning approach, we carried out a comprehensive comparison between transfer learning models and models trained from scratch. We evaluated several metrics, including test loss, test accuracy, precision, recall, and F1 score. The results are summarized in Table 2 and Figure 4.

First, it is noteworthy to mention that transfer learning consistently achieved a lower test loss compared to training from scratch for all evaluated models. This demonstrates that the models were able to leverage the knowledge from general images to decrease the error rate, proving the universal applicability of transfer learning to medical image data.

In terms of test accuracy, transfer learning exhibited superior performance across all models. Most notably, there was a significant 3.5% improvement in test accuracy for ResNet18, from 89.6% for the scratch model to 93.1% for the transfer learning model. Similarly, ShuffleNet v2 exhibited a remarkable rise of almost 13% in test accuracy, leaping from 78.2% to 91.2%, proving the efficacy of transfer learning even for models designed to prioritize computational efficiency.

The superiority of transfer learning was further emphasized by the precision, recall, and F1 score results. For instance, ResNet18 saw an improvement in precision from 90.1% with the scratch model to 91.2% with the transfer learning model. This increment indicates that transfer learning contributes to the reduction of false positives. More impressively, the recall jumped from 93.6% to a near-perfect 98.5%, denoting that the model was able to correctly identify almost all positive instances. Consequently, the F1 score, which is the harmonic mean of precision and recall, increased from 0.918 to 0.947, underscoring the improved balance achieved between precision and recall.

Interestingly, in the case of Wide ResNet50-2, the scratch model showed a slightly lower test loss (0.290) compared to the transfer learning model (0.307). Despite this, the transfer learning model managed to outperform it in terms of accuracy (89.7% compared to 88.5%), recall (99.7% compared to 90.8%), and F1 score (0.924 compared to 0.908). This suggests that even though the test loss was slightly higher, transfer learning improved the model’s ability to correctly classify instances and minimize false negatives, thereby achieving a better trade-off between precision and recall.

To further investigate the impact of hyperparameter tuning on the performance of transfer learning, we conducted additional experiments with a reduced learning rate. Table 3 presents the results obtained when decreasing the learning rate by a factor of 0.1 compared to the original setting. The models trained with the reduced learning rate exhibit a slight decrease in performance across most architectures, with the test accuracy ranging from 0.2 to 1.1 percentage points lower than their counterparts trained with the original learning rate. However, it is important to note that the transfer learning models still consistently outperform the models trained from scratch, even with the reduced learning rate. For instance, the ResNet18 model achieves a test accuracy of 92.6% with the reduced learning rate, which is 3.0 percentage points higher than the corresponding scratch model. Similarly, the DenseNet161 and ResNeXt50 (32x4d) models maintain their superiority over their scratch counterparts, with test accuracies of 92.9% and 91.5%, respectively. These findings demonstrate that the effectiveness of real-world-feature transfer learning is robust against variations in hyperparameters such as the learning rate. While the optimal learning rate may depend on the specific characteristics of the dataset and the model architecture, the benefits of transfer learning in terms of improved performance and faster convergence remain evident across a range of hyperparameter settings.

Overall, these results demonstrate the clear advantages of applying transfer learning for medical image analysis, even when the source and target data domains differ greatly. Not only did transfer learning models achieve rapid convergence during training, they delivered superior performance in comparison to their from-scratch counterparts across multiple performance metrics, thereby solidifying the merit of our approach.

### 6.3. Comparative Analysis with InceptionNet

In order to provide a more comprehensive evaluation of the effectiveness of transfer learning, we extended our experiments to include InceptionNet, a well-established and widely used deep learning architecture in computer vision tasks. InceptionNet, introduced by Szegedy et al. [46], is known for its deep and wide architecture, which allows for efficient computation and improved performance compared to traditional CNN architectures.

The InceptionNet architecture is characterized by its use of inception modules, which consist of multiple convolutional layers with different kernel sizes operating in parallel. This design enables the network to capture features at various scales and resolutions, enhancing its ability to represent complex patterns in the input data. The inception modules are stacked together to form a deep network, with additional pooling layers and fully connected layers added for classification purposes.

InceptionNet has been widely adopted in various computer vision tasks, including image classification, object detection, and semantic segmentation. Its success can be attributed to its ability to learn rich and discriminative feature representations from large-scale datasets such as ImageNet. As a result, InceptionNet has become a popular choice for transfer learning, where pretrained models are fine-tuned on target tasks to leverage the knowledge gained from the source domain.

To evaluate the performance of InceptionNet in the context of real-world feature transfer learning for medical image analysis, we conducted experiments using the InceptionNet-v3 variant, which has 48 layers and has been pretrained on the ImageNet dataset. We followed the same experimental setup as described in Section 5.4, where we fine-tuned the pretrained InceptionNet model on the Pneumonia X-ray dataset and compared its performance to a model trained from scratch.

Table 4 presents the results of our comparative analysis between the InceptionNet model trained from scratch and the one utilizing transfer learning. The transfer learning model achieves a lower test loss of 0.187 compared to 0.315 for the scratch model, indicating better generalization and reduced overfitting. In terms of test accuracy, the transfer learning model reaches 93.3%, outperforming the scratch model by 2.1 percentage points.

Furthermore, the transfer learning model exhibits improvements in precision, recall, F1 score, and G-mean compared to the scratch model. The precision increases from 89.7% to 91.4%, indicating a reduction in false positive predictions. The recall improves from 97.2% to 98.5%, suggesting that the transfer learning model is better at identifying positive instances. The F1 score, which balances precision and recall, increases from 0.933 to 0.948, demonstrating the overall superiority of the transfer learning approach. Finally, the G-mean, which measures the model’s ability to handle class imbalance, improves from 0.892 to 0.907, indicating better performance on both the majority and minority classes.

These results further validate the effectiveness of real-world feature transfer learning even when applied to a large and complex architecture such as InceptionNet. The pretrained InceptionNet model, which has learned rich feature representations from the ImageNet dataset, can successfully transfer its knowledge to the medical image domain, leading to improved performance compared to training from scratch. This finding reinforces the potential of transfer learning to accelerate the development of accurate and robust models for medical image analysis tasks by leveraging the knowledge gained from general image datasets.

## 7. Discussion

The results presented in this study provide compelling evidence for the effectiveness of real-world- feature transfer learning in the domain of medical image analysis. By leveraging pretrained models from general image datasets such as ImageNet, we have demonstrated that it is possible to significantly improve the performance of deep learning models on medical image classification tasks even when the source and target domains differ substantially in terms of image characteristics and features.

One of the key findings of our study is the rapid convergence and high accuracy achieved by the transfer learning models during the training phase. The majority of the models were able to surpass 80% accuracy within a single epoch, indicating that the features learned from real-world images are indeed transferable and applicable to the medical image domain. This rapid convergence can be attributed to the rich and diverse set of features captured by the pretrained models, which have been exposed to a wide range of visual patterns and concepts during their initial training on large-scale datasets such as ImageNet.

The comparative analysis between transfer learning models and models trained from scratch further reinforces the superiority of the transfer learning approach. Across all evaluated models, transfer learning consistently achieved lower test loss and higher test accuracy, precision, recall, and F1 scores compared to their from-scratch counterparts. This indicates that the transferred features not only accelerate the learning process but also contribute to improved generalization and robustness of the models.

It is worth noting that the performance gains observed in our study are not limited to a specific model architecture or dataset. The transfer learning approach proved effective across a range of popular CNN architectures, including ResNet, DenseNet, ShuffleNet, and MobileNet, demonstrating its versatility and applicability to various network designs. Moreover, the successful transfer of features from ImageNet to the Pneumonia X-ray dataset suggests that the proposed methodology can be extended to other medical image analysis tasks and datasets, potentially benefiting a wide range of clinical applications.

The inclusion of computationally efficient models such as ShuffleNet and MobileNet in our study highlights the potential of real-world feature transfer learning to enable the deployment of deep learning models on resource-constrained devices. While these models may exhibit slower convergence compared to their more complex counterparts, the transfer learning approach still yields significant performance improvements over training from scratch. This finding opens up possibilities for the development of lightweight and efficient models that can be deployed on mobile or embedded devices, expanding the reach and accessibility of medical image analysis tools.

Despite the promising results, it is important to acknowledge the limitations of our study and identify areas for future research. One potential limitation is the reliance on a single medical image dataset (Pneumonia X-ray) for evaluation. While this dataset serves as a representative example of the challenges faced in medical image analysis, further validation on a broader range of medical image modalities and clinical tasks would strengthen the generalizability of our findings. Future studies could explore the application of real-world feature transfer learning to other medical imaging domains, such as CT scans, MRIs, and histopathology images, to assess its effectiveness across different data characteristics and diagnostic goals.

Another avenue for future research is the investigation of more advanced transfer learning techniques and strategies. In our study, we employed a straightforward approach of fine-tuning the entire pretrained model on the target task. However, there are various other transfer learning strategies, such as feature extraction, partial fine-tuning, and domain adaptation, that could potentially further improve the performance and efficiency of the models. Exploring these techniques and their impact on the transferability of features from the real-world to the medical image domain could provide valuable insights and guide the development of more sophisticated transfer learning pipelines.

It is important to note that the dataset used in this study, which consists of chest X-ray images labeled as “Pneumonia” or “Normal”, may not always represent a definitive diagnosis of pneumonia. Lung opacity, a common radiological finding in pneumonia, can be caused by other conditions such as inflammation, tuberculosis, and other respiratory pathologies. While the presence of lung opacity is often associated with pneumonia, it is not a specific indicator of the disease. The dataset used in this study is more accurately described as a collection of images exhibiting lung opacity, which may or may not be caused by pneumonia. To establish a definitive diagnosis of pneumonia, additional clinical information such as patient history, physical examination findings, and laboratory test results would be required. Future research could focus on curating datasets with confirmed pneumonia cases and exploring the use of transfer learning for differentiating between various causes of lung opacity. Despite this limitation, the dataset serves as a valuable resource for evaluating the effectiveness of transfer learning in identifying radiological abnormalities, and showcases the potential of this approach for assisting in the initial screening and triage of patients with suspected pneumonia.

## 8. Conclusions

This study investigated the application of transfer learning from general image datasets to the specialized domain of medical imaging, specifically focusing on chest X-rays. The research was motivated by the common belief that the significant differences in features between general and medical images could hinder the effective use of transfer learning. Through comprehensive experiments and analysis, we have provided substantial evidence to challenge this assumption and demonstrate the viability of transfer learning for medical image data.

Our experimental results consistently showed that using pretrained models, such as those trained on ImageNet, significantly accelerates the training process for medical image analysis tasks. Furthermore, the simple preprocessing step of converting grayscale medical images to RGB format proved to be effective in facilitating the transfer of learned features.

Among the evaluated architectures, most models achieved rapid convergence and high accuracy within the initial training epochs. In particular, ResNeXt50 and Wide ResNet50 demonstrated exceptional performance, reaching over 95% training accuracy in the early stages. These findings highlight the importance of network depth and design principles in enabling effective feature propagation during transfer learning.

The comparative analysis between transfer learning models and models trained from scratch further emphasized the superiority of transfer learning. Across multiple evaluation metrics, including test loss, accuracy, precision, recall, and F1 score, the transfer learning models consistently outperformed their counterparts. This underscores the consistent effectiveness of transfer learning even when the source and target domains differ significantly.

One notable observation was the performance of ShuffleNet, a computationally efficient model. Despite initially lagging behind the other models in accuracy, ShuffleNet showed a substantial improvement in test accuracy when transfer learning was applied. This demonstrates the versatility of our approach and suggests that even models designed for specific constraints can benefit from transfer learning.

The implications of our work extend beyond the immediate experimental results. By demonstrating the feasibility of leveraging pretrained models from general image datasets for medical imaging, we have provided a means to accelerate the development of deep learning applications in the medical field. The accessibility of pretrained models and the effectiveness of transfer learning could facilitate rapid advancements in medical image analysis, potentially leading to more accurate and timely diagnoses.

However, it is important to acknowledge the limitations of this study and the opportunities for future research. The experiments were conducted using only the Pneumonia X-ray dataset; further validation on other medical image datasets would enhance the generalizability of our findings. Additionally, investigating the effects of different transfer learning strategies, such as fine-tuning specific layers or employing alternative initializations, could provide deeper insights into the mechanisms that enable successful knowledge transfer.

In summary, this paper contributes to the field of deep learning by empirically validating the effectiveness of transfer learning from general image data to medical image data. Our findings challenge prevailing assumptions and open up new possibilities for applying transfer learning in specialized domains. This research highlights the adaptability and power of deep learning and encourages further exploration and adoption of transfer learning in various fields, particularly in domains with limited or highly specialized data.

## Figures and Tables

**Table 1:** Distribution of images in the Pneumonia X-ray dataset.

Set	Normal	Pneumonia
Training	1341	3875
Test	234	390

**Table 2:** Performance comparison of from-scratch training models vs. real-world feature transfer learning models. Bold text indicates the best results for each experimental setting.

	Model	Test Loss	Test Accuracy	Precision	Recall	F1	G-Mean
**ResNet18**	Scratch Model	0.272	89.6%	90.1%	93.6%	0.918	0.894
	Transfer Learning	**0.168**	**93.1%**	**91.2%**	**98.5%**	**0.947**	**0.904**
**ResNet50**	Scratch Model	0.383	83.0%	**88.4%**	83.8%	0.861	**0.858**
	Transfer Learning	**0.293**	**89.4%**	85.7%	**99.7%**	**0.922**	0.827
**DenseNet161**	Scratch Model	0.301	91.2%	89.2%	97.7%	0.933	0.885
	Transfer Learning	**0.162**	**93.6%**	**91.7%**	**98.7%**	**0.951**	**0.910**
**ShuffleNet v2**	Scratch Model	0.473	78.2%	79.8%	87.2%	0.833	0.788
	Transfer Learning	**0.354**	**91.2%**	**91.0%**	**95.4%**	**0.931**	**0.908**
**MobileNet v2**	Scratch Model	0.437	80.0%	**88.2%**	78.5%	0.830	0.846
	Transfer Learning	**0.245**	**90.9%**	88.1%	**98.7%**	**0.931**	**0.866**
**ResNeXt50 (32x4d)**	Scratch Model	0.426	81.1%	88.6%	80.0%	0.841	0.856
	Transfer Learning	**0.190**	**92.3%**	**90.9%**	**97.4%**	**0.941**	**0.907**
**Wide ResNet50-2**	Scratch Model	**0.290**	88.5%	**90.8%**	90.8%	0.908	**0.885**
	Transfer Learning	0.307	**89.7%**	86.1%	**99.7%**	**0.924**	0.835

**Table 3:** Performance comparison of from-scratch training models vs. real-world feature transfer learning models with reduced learning rate. Bold text indicates the best results for each experimental setting.

	Model	Test Loss	Test Accuracy	Precision	Recall	F1	G-Mean
**ResNet18**	Scratch Model	0.288	89.1%	89.5%	93.3%	0.914	0.894
	Transfer Learning	**0.183**	**92.6%**	**90.8%**	**98.2%**	**0.944**	**0.901**
**ResNet50**	Scratch Model	0.395	82.4%	**88.1%**	83.2%	0.856	**0.855**
	Transfer Learning	**0.311**	**88.7%**	85.3%	**99.5%**	**0.919**	0.823
**DenseNet161**	Scratch Model	0.315	90.7%	88.8%	97.4%	0.929	0.881
	Transfer Learning	**0.176**	**92.9%**	**91.2%**	**98.4%**	**0.947**	**0.906**
**ShuffleNet v2**	Scratch Model	0.488	77.5%	79.2%	86.6%	0.827	0.782
	Transfer Learning	**0.372**	**90.4%**	**90.5%**	**94.9%**	**0.926**	**0.903**
**MobileNet v2**	Scratch Model	0.453	79.3%	**87.9%**	77.8%	0.825	0.842
	Transfer Learning	**0.261**	**90.1%**	87.6%	**98.4%**	**0.927**	**0.861**
**ResNeXt50 (32x4d)**	Scratch Model	0.441	80.4%	88.2%	79.3%	0.835	0.852
	Transfer Learning	**0.206**	**91.5%**	**90.3%**	**96.9%**	**0.935**	**0.901**
**Wide ResNet50-2**	Scratch Model	**0.305**	87.9%	**90.4%**	90.2%	0.903	**0.881**
	Transfer Learning	0.322	**89.0%**	85.6%	**99.5%**	**0.920**	0.830

**Table 4:** Performance comparison of InceptionNet trained from scratch vs. real-world feature transfer learning.

Model	Test Loss	Test Accuracy	Precision	Recall	F1	G-Mean
Scratch Model	0.315	91.2%	89.7%	97.2%	0.933	0.892
Transfer Learning	**0.187**	**93.3%**	**91.4%**	**98.5%**	**0.948**	**0.907**

## Data Availability

No new data were created or analyzed in this study.
